# Poly[tris­[μ_2_-2-(pyrazol-1-yl)pyrazine]hexa-μ_1,3_-thio­cyanato-tricadmium(II)]

**DOI:** 10.1107/S1600536808032285

**Published:** 2008-10-11

**Authors:** Lin Yan Yang, Jing Min Shi

**Affiliations:** a Department of Chemistry, Shandong Normal University, Jinan 250014, People’s Republic of China

## Abstract

The asymmetric unit of the title crystal structure, [Cd_3_(NCS)_6_(C_7_H_6_N_4_)_2_]_*n*_, contains two independent Cd^II^ ions, one of which is located on a crystallographic inversion center. Each independent Cd^II^ ion is in a slightly distorted octa­hedral coordination environment, but the disortion from ideally octa­hedral is greater in the environment of the Cd^II^ ion on a general position. Both thio­cyanate ligands act as bridges connecting independent Cd^II^ ions, and the 2-(pyrazol-1-yl)pyrazine ligands chelate one Cd^II^ ion in a bidentate mode while the remaining N atom of the pyrazine ring coordinates to a symmetry-related Cd^II^ ion, forming a two-dimensional structure parallel to (211).

## Related literature

For background information, see: Shi, Sun, Liu *et al.* (2006 ▶); Shi, Sun, Zhang *et al.* (2006 ▶). 
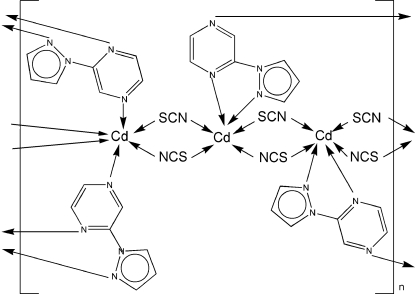


## Experimental

### 

#### Crystal data

[Cd_3_(NCS)_6_(C_7_H_6_N_4_)_2_]
*M* *_r_* = 978.00
Triclinic, 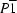

*a* = 7.0309 (9) Å
*b* = 8.6178 (12) Å
*c* = 13.7373 (18) Å
α = 87.889 (2)°
β = 85.173 (2)°
γ = 68.060 (2)°
*V* = 769.32 (18) Å^3^
*Z* = 1
Mo *K*α radiation
μ = 2.50 mm^−1^
*T* = 298 (2) K
0.38 × 0.16 × 0.10 mm

#### Data collection

Bruker SMART APEX CCD diffractometer
Absorption correction: multi-scan (*SADABS*; Sheldrick, 1996 ▶) *T*_min_ = 0.450, *T*_max_ = 0.788
4043 measured reflections
2805 independent reflections
2625 reflections with *I* > 2σ(*I*)
*R*_int_ = 0.013

#### Refinement

*R*[*F* ^2^ > 2σ(*F* ^2^)] = 0.022
*wR*(*F* ^2^) = 0.056
*S* = 1.06
2805 reflections
196 parameters
H-atom parameters constrained
Δρ_max_ = 0.61 e Å^−3^
Δρ_min_ = −0.51 e Å^−3^

### 

Data collection: *SMART* (Bruker, 1997 ▶); cell refinement: *SAINT* (Bruker, 1997 ▶); data reduction: *SAINT*; program(s) used to solve structure: *SHELXTL* (Sheldrick, 2008 ▶); program(s) used to refine structure: *SHELXTL*; molecular graphics: *SHELXTL*; software used to prepare material for publication: *SHELXTL*.

## Supplementary Material

Crystal structure: contains datablocks I, global. DOI: 10.1107/S1600536808032285/lh2704sup1.cif

Structure factors: contains datablocks I. DOI: 10.1107/S1600536808032285/lh2704Isup2.hkl

Additional supplementary materials:  crystallographic information; 3D view; checkCIF report

## Figures and Tables

**Table 1:** Selected bond lengths (Å)

Cd1—N2	2.286 (3)
Cd1—N7^i^	2.426 (2)
Cd1—S2	2.6832 (8)
Cd2—N1^ii^	2.244 (2)
Cd2—N3	2.303 (3)
Cd2—N5	2.385 (2)
Cd2—N4	2.436 (2)
Cd2—S1	2.6603 (9)
Cd2—S3	2.7427 (8)

Symmetry codes: (i) 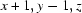; (ii) 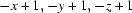.

**Fig. 1.**
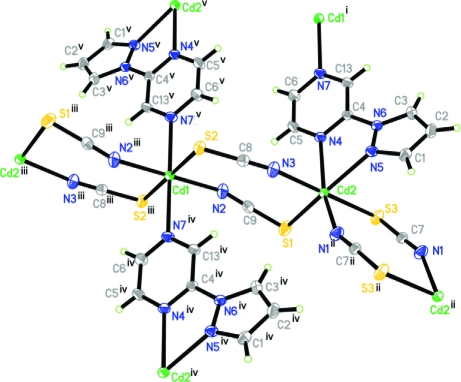
Coordination around the two independent CdII ions in (I) with the atom-numbering scheme. Displacement ellipsoids are drawn at the 30% probability level [symmetry codes: (i) x-1, y+1, z (ii) -x+1, -y+1, -z+1 (iii) -x+1, -y, -z (iv) x+1, y-1, z (v) -x, -y+1, -z].

**Fig. 2.**
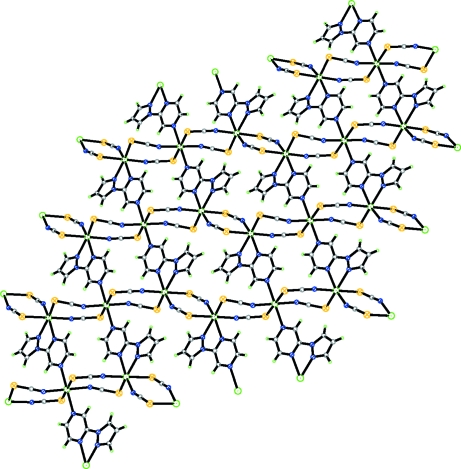
Part of the two-dimensional sheet structure of (I).

## supplementary crystallographic information

### Comment

For a considerable time, interest has focused on polymeric coordination compounds because such new coordination polymers may afford new materials with useful properties, such as catalytic activity, micro-porosity, electrical conductivity, non-linear optical activity and  magnetic coupling behavior. The thiocyanide anion is a very common bridging ligand and many muti-nuclear complexes containing this ligand have been reported. Some of these complexes exhibit interesting magnetic coupling properties (Shi, Sun, Liu *et al.*, 2006; Shi, Sun, Zhang *et al.*, 2006)). The 2-(pyrazole-1-yl)-pyrazine molecule can act as a bridge ligand due to its structural character and up till now no crystal structures of complexes with this ligand have been reported. Under the motivation of preparing new coordination polymers containing mixed bridging ligands, we have synthesized the title coordination polymer and herein we report its crystal structure (I).

Fig. 1 shows the coordination around each independent Cd^II^ ion. Atom Cd1 is located on a crystallographic inversion center. In the crystal structure thiocyanade anions act as bridging ligands and connect symmetry related Cd^II^ ions [with Cd···Cd = 5.8122 (6)Å for Cd1···Cd2 and 5.7411 (7) Å for Cd2···Cd2^ii^; symmetry code: (ii) -x+1, -y+1, -z+1] forming an eight-membered ring which acts as a repeat unit of the structure in one-dimension [Fig. 2]. The 2-(pyrazole-1-yl)-pyrazine ligand functions as a tridentate bridging ligand and coordinates to symmetry related Cd^II^ ions [with a Cd···Cd separation of 7.6144 (7)Å] connecting the structure further into two-dimensions. Figure 2 also shows that in the two-dimensional structure there are two different types of rings formed by the 2-(pyrazole-1-yl)-pyrazine bridging ligand. An 18-membered ring consists of four Cd^II^ ions, two thiocyanato ligands and two 2-(pyrazole-1-yl)-pyrazine bridging ligands while a 26-membered ring consists of two 2-(pyrazole-1-yl)-pyrazine bridging ligands, four thiocyanade ligands and six Cd^II^ ions. The 18-membered rings and the 26-membered rings are arranged alternately in the two-dimensional structure.

### Experimental

7 ml 3-(pyrazole-2-yloxy)-pyridine (0.0365 g, 0.250 mmol) methanol solution, 7 ml C d(ClO_4_)_2_6H_2_O (0.1048 g, 0.250 mmol) H_2_O solution and 4 ml NaSCN (0.0405 g, 0.500 mmol) H_2_O solution were mixed together and stirred for a few minutes. Colorless single crystals were obtained after allowing the filtrate to stand at room temperature for four months.

### Refinement

All H atoms were placed in calculated positions and refined as riding with (C—H = 0.93 Å) and *U*_iso_ = 1.2*U*_eq_(C).

### Crystal data

**Table:**

[Cd_3_(NCS)_6_(C_7_H_6_N_4_)_2_]	*Z* = 1
*M**_r_* = 978.00	*F*(000) = 470
Triclinic, *P*1	*D*_x_ = 2.111 Mg m^−^^3^
Hall symbol: -P 1	Mo *K*α radiation, λ = 0.71073 Å
*a* = 7.0309 (9) Å	Cell parameters from 3404 reflections
*b* = 8.6178 (12) Å	θ = 2.6–28.2°
*c* = 13.7373 (18) Å	µ = 2.50 mm^−^^1^
α = 87.889 (2)°	*T* = 298 K
β = 85.173 (2)°	Bar, colorless
γ = 68.060 (2)°	0.38 × 0.16 × 0.10 mm
*V* = 769.32 (18) Å^3^	

### Data collection

**Table:**

Bruker SMART APEX CCD diffractometer	2805 independent reflections
Radiation source: fine-focus sealed tube	2625 reflections with *I* > 2σ(*I*)
graphite	*R*_int_ = 0.013
φ and ω scans	θ_max_ = 25.5°, θ_min_ = 2.6°
Absorption correction: multi-scan (SADABS; Sheldrick, 1996)	*h* = −4→8
*T*_min_ = 0.450, *T*_max_ = 0.788	*k* = −10→10
4043 measured reflections	*l* = −15→16

### Refinement

**Table:**

Refinement on *F*^2^	Primary atom site location: structure-invariant direct methods
Least-squares matrix: full	Secondary atom site location: difference Fourier map
*R*[*F*^2^ > 2σ(*F*^2^)] = 0.022	Hydrogen site location: inferred from neighbouring sites
*wR*(*F*^2^) = 0.056	H-atom parameters constrained
*S* = 1.06	*w* = 1/[σ^2^(*F*_o_^2^) + (0.0271*P*)^2^ + 0.3586*P*] where *P* = (*F*_o_^2^ + 2*F*_c_^2^)/3
2805 reflections	(Δ/σ)_max_ = 0.001
196 parameters	Δρ_max_ = 0.61 e Å^−^^3^
0 restraints	Δρ_min_ = −0.51 e Å^−^^3^

### Special details

**Table:**

Geometry. All e.s.d.'s (except the e.s.d. in the dihedral angle between two l.s. planes) are estimated using the full covariance matrix. The cell e.s.d.'s are taken into account individually in the estimation of e.s.d.'s in distances, angles and torsion angles; correlations between e.s.d.'s in cell parameters are only used when they are defined by crystal symmetry. An approximate (isotropic) treatment of cell e.s.d.'s is used for estimating e.s.d.'s involving l.s. planes.
Refinement. Refinement of *F*^2^ against ALL reflections. The weighted *R*-factor *wR* and goodness of fit *S* are based on *F*^2^, conventional *R*-factors *R* are based on *F*, with *F* set to zero for negative *F*^2^. The threshold expression of *F*^2^ > σ(*F*^2^) is used only for calculating *R*-factors(gt) *etc*. and is not relevant to the choice of reflections for refinement. *R*-factors based on *F*^2^ are statistically about twice as large as those based on *F*, and *R*- factors based on ALL data will be even larger.

### Fractional atomic coordinates and isotropic or equivalent isotropic displacement parameters (Å^2^)

**Table:**

	*x*	*y*	*z*	*U*_iso_*/*U*_eq_	
C1	0.0009 (4)	0.7101 (4)	0.49099 (19)	0.0358 (6)	
H1	0.0564	0.6478	0.5456	0.043*	
C2	−0.1423 (5)	0.8730 (4)	0.4948 (2)	0.0402 (7)	
H2	−0.1990	0.9378	0.5502	0.048*	
C3	−0.1821 (5)	0.9178 (4)	0.4006 (2)	0.0383 (7)	
H3	−0.2725	1.0200	0.3788	0.046*	
C4	−0.0578 (4)	0.7640 (3)	0.24350 (18)	0.0260 (5)	
C5	0.1109 (5)	0.6225 (4)	0.1068 (2)	0.0381 (7)	
H5	0.2239	0.5397	0.0758	0.046*	
C6	−0.0462 (5)	0.7197 (4)	0.0532 (2)	0.0388 (7)	
H6	−0.0368	0.7019	−0.0137	0.047*	
C7	0.5004 (4)	0.6931 (3)	0.4694 (2)	0.0312 (6)	
C8	0.2476 (4)	0.1598 (3)	0.21816 (19)	0.0309 (6)	
C9	0.6045 (4)	0.3004 (4)	0.1046 (2)	0.0369 (7)	
C13	−0.2187 (4)	0.8625 (3)	0.18960 (19)	0.0311 (6)	
H13	−0.3319	0.9454	0.2206	0.037*	
Cd1	0.5000	0.0000	0.0000	0.02922 (9)	
Cd2	0.35673 (3)	0.47371 (2)	0.314014 (13)	0.02935 (8)	
N1	0.5156 (4)	0.6693 (3)	0.55123 (18)	0.0419 (6)	
N2	0.5526 (4)	0.2304 (3)	0.04926 (19)	0.0445 (6)	
N3	0.2614 (4)	0.2620 (3)	0.26533 (18)	0.0438 (6)	
N4	0.1055 (3)	0.6441 (3)	0.20341 (16)	0.0298 (5)	
N5	0.0489 (3)	0.6539 (3)	0.40006 (16)	0.0311 (5)	
N6	−0.0646 (3)	0.7848 (3)	0.34443 (15)	0.0292 (5)	
N7	−0.2124 (3)	0.8392 (3)	0.09389 (16)	0.0325 (5)	
S1	0.67535 (14)	0.40534 (14)	0.18169 (7)	0.0617 (3)	
S2	0.22912 (12)	0.01127 (9)	0.15191 (5)	0.03591 (17)	
S3	0.47355 (13)	0.73397 (10)	0.35252 (5)	0.04143 (19)	

### Atomic displacement parameters (Å^2^)

**Table:**

	*U*^11^	*U*^22^	*U*^33^	*U*^12^	*U*^13^	*U*^23^
C1	0.0406 (16)	0.0454 (16)	0.0255 (14)	−0.0200 (14)	−0.0055 (12)	0.0002 (12)
C2	0.0428 (17)	0.0477 (17)	0.0322 (15)	−0.0193 (14)	0.0024 (13)	−0.0120 (13)
C3	0.0397 (16)	0.0325 (15)	0.0388 (16)	−0.0084 (13)	−0.0025 (13)	−0.0082 (12)
C4	0.0233 (13)	0.0304 (13)	0.0244 (13)	−0.0100 (11)	−0.0017 (10)	0.0002 (10)
C5	0.0332 (15)	0.0398 (16)	0.0282 (14)	0.0017 (13)	−0.0024 (12)	−0.0046 (12)
C6	0.0380 (16)	0.0445 (16)	0.0243 (14)	−0.0040 (13)	−0.0039 (12)	−0.0001 (12)
C7	0.0316 (14)	0.0281 (13)	0.0344 (16)	−0.0105 (11)	−0.0082 (12)	−0.0009 (11)
C8	0.0312 (15)	0.0360 (15)	0.0247 (13)	−0.0121 (12)	−0.0023 (11)	0.0042 (12)
C9	0.0312 (15)	0.0371 (15)	0.0368 (16)	−0.0075 (13)	0.0061 (12)	−0.0059 (13)
C13	0.0250 (13)	0.0337 (14)	0.0285 (14)	−0.0036 (11)	−0.0035 (11)	0.0007 (11)
Cd1	0.02909 (16)	0.03095 (15)	0.02367 (15)	−0.00539 (12)	−0.00732 (11)	−0.00178 (11)
Cd2	0.02855 (12)	0.03169 (12)	0.02402 (12)	−0.00559 (9)	−0.00708 (8)	−0.00237 (8)
N1	0.0545 (16)	0.0386 (14)	0.0348 (14)	−0.0171 (12)	−0.0183 (12)	0.0057 (11)
N2	0.0539 (17)	0.0435 (15)	0.0377 (14)	−0.0203 (13)	0.0023 (12)	−0.0115 (12)
N3	0.0551 (17)	0.0472 (15)	0.0326 (13)	−0.0228 (13)	−0.0010 (12)	−0.0081 (12)
N4	0.0251 (11)	0.0327 (12)	0.0280 (12)	−0.0062 (10)	−0.0057 (9)	0.0008 (9)
N5	0.0288 (12)	0.0353 (12)	0.0266 (11)	−0.0088 (10)	−0.0045 (9)	0.0028 (9)
N6	0.0260 (11)	0.0327 (12)	0.0263 (11)	−0.0072 (9)	−0.0057 (9)	−0.0008 (9)
N7	0.0271 (12)	0.0367 (12)	0.0283 (12)	−0.0053 (10)	−0.0056 (10)	0.0032 (10)
S1	0.0419 (5)	0.0957 (7)	0.0566 (5)	−0.0359 (5)	0.0139 (4)	−0.0407 (5)
S2	0.0423 (4)	0.0402 (4)	0.0301 (4)	−0.0209 (3)	−0.0007 (3)	−0.0051 (3)
S3	0.0571 (5)	0.0523 (5)	0.0274 (4)	−0.0342 (4)	−0.0067 (3)	0.0023 (3)

### Geometric parameters (Å, °)

**Table:**

C1—N5	1.327 (3)	C9—N2	1.148 (4)
C1—C2	1.388 (4)	C9—S1	1.639 (3)
C1—H1	0.9300	C13—N7	1.332 (3)
C2—C3	1.359 (4)	C13—H13	0.9300
C2—H2	0.9300	Cd1—N2	2.286 (3)
C3—N6	1.357 (3)	Cd1—N2^i^	2.286 (3)
C3—H3	0.9300	Cd1—N7^ii^	2.426 (2)
C4—N4	1.319 (3)	Cd1—N7^iii^	2.426 (2)
C4—C13	1.388 (4)	Cd1—S2^i^	2.6832 (8)
C4—N6	1.399 (3)	Cd1—S2	2.6832 (8)
C5—N4	1.342 (3)	Cd2—N1^iv^	2.244 (2)
C5—C6	1.365 (4)	Cd2—N3	2.303 (3)
C5—H5	0.9300	Cd2—N5	2.385 (2)
C6—N7	1.331 (4)	Cd2—N4	2.436 (2)
C6—H6	0.9300	Cd2—S1	2.6603 (9)
C7—N1	1.142 (3)	Cd2—S3	2.7427 (8)
C7—S3	1.643 (3)	N1—Cd2^iv^	2.244 (2)
C8—N3	1.150 (4)	N5—N6	1.364 (3)
C8—S2	1.647 (3)	N7—Cd1^v^	2.426 (2)
			
N5—C1—C2	111.8 (3)	N7^ii^—Cd1—S2	91.66 (6)
N5—C1—H1	124.1	N7^iii^—Cd1—S2	88.34 (6)
C2—C1—H1	124.1	S2^i^—Cd1—S2	180
C3—C2—C1	105.6 (3)	N1^iv^—Cd2—N3	91.59 (9)
C3—C2—H2	127.2	N1^iv^—Cd2—N5	93.71 (9)
C1—C2—H2	127.2	N3—Cd2—N5	102.07 (9)
N6—C3—C2	106.9 (3)	N1^iv^—Cd2—N4	159.57 (9)
N6—C3—H3	126.6	N3—Cd2—N4	83.77 (9)
C2—C3—H3	126.6	N5—Cd2—N4	68.04 (7)
N4—C4—C13	122.2 (2)	N1^iv^—Cd2—S1	105.57 (8)
N4—C4—N6	116.7 (2)	N3—Cd2—S1	94.46 (7)
C13—C4—N6	121.1 (2)	N5—Cd2—S1	154.21 (6)
N4—C5—C6	121.4 (3)	N4—Cd2—S1	94.63 (6)
N4—C5—H5	119.3	N1^iv^—Cd2—S3	93.51 (7)
C6—C5—H5	119.3	N3—Cd2—S3	174.25 (7)
N7—C6—C5	121.9 (3)	N5—Cd2—S3	80.27 (6)
N7—C6—H6	119.0	N4—Cd2—S3	92.33 (6)
C5—C6—H6	119.0	S1—Cd2—S3	81.60 (3)
N1—C7—S3	178.1 (3)	C7—N1—Cd2^iv^	156.4 (2)
N3—C8—S2	179.1 (3)	C9—N2—Cd1	151.9 (3)
N2—C9—S1	178.3 (3)	C8—N3—Cd2	160.7 (2)
N7—C13—C4	120.5 (2)	C4—N4—C5	116.7 (2)
N7—C13—H13	119.7	C4—N4—Cd2	116.51 (17)
C4—C13—H13	119.7	C5—N4—Cd2	126.65 (18)
N2—Cd1—N2^i^	180	C1—N5—N6	104.4 (2)
N2—Cd1—N7^ii^	85.94 (9)	C1—N5—Cd2	134.13 (19)
N2^i^—Cd1—N7^ii^	94.06 (9)	N6—N5—Cd2	112.89 (15)
N2—Cd1—N7^iii^	94.06 (9)	C3—N6—N5	111.4 (2)
N2^i^—Cd1—N7^iii^	85.94 (9)	C3—N6—C4	129.3 (2)
N7^ii^—Cd1—N7^iii^	180	N5—N6—C4	119.2 (2)
N2—Cd1—S2^i^	86.38 (7)	C6—N7—C13	117.2 (2)
N2^i^—Cd1—S2^i^	93.62 (7)	C6—N7—Cd1^v^	121.71 (18)
N7^ii^—Cd1—S2^i^	88.34 (6)	C13—N7—Cd1^v^	120.98 (18)
N7^iii^—Cd1—S2^i^	91.66 (6)	C9—S1—Cd2	98.65 (11)
N2—Cd1—S2	93.62 (7)	C8—S2—Cd1	101.07 (10)
N2^i^—Cd1—S2	86.38 (7)	C7—S3—Cd2	96.52 (10)
			
N5—C1—C2—C3	−0.3 (4)	S1—Cd2—N5—C1	−110.8 (3)
C1—C2—C3—N6	−0.3 (3)	S3—Cd2—N5—C1	−64.9 (3)
N4—C5—C6—N7	−0.5 (5)	N1^iv^—Cd2—N5—N6	169.76 (17)
N4—C4—C13—N7	−0.5 (4)	N3—Cd2—N5—N6	−97.82 (18)
N6—C4—C13—N7	−178.6 (2)	N4—Cd2—N5—N6	−19.70 (16)
N7^ii^—Cd1—N2—C9	−30.1 (5)	S1—Cd2—N5—N6	30.9 (3)
N7^iii^—Cd1—N2—C9	149.9 (5)	S3—Cd2—N5—N6	76.83 (17)
S2^i^—Cd1—N2—C9	−118.7 (5)	C2—C3—N6—N5	0.7 (3)
S2—Cd1—N2—C9	61.3 (5)	C2—C3—N6—C4	176.0 (3)
N1^iv^—Cd2—N3—C8	−124.3 (8)	C1—N5—N6—C3	−0.9 (3)
N5—Cd2—N3—C8	141.6 (8)	Cd2—N5—N6—C3	−153.58 (19)
N4—Cd2—N3—C8	75.7 (8)	C1—N5—N6—C4	−176.7 (2)
S1—Cd2—N3—C8	−18.5 (8)	Cd2—N5—N6—C4	30.6 (3)
C13—C4—N4—C5	0.9 (4)	N4—C4—N6—C3	162.2 (3)
N6—C4—N4—C5	179.1 (3)	C13—C4—N6—C3	−19.5 (4)
C13—C4—N4—Cd2	−175.6 (2)	N4—C4—N6—N5	−22.8 (3)
N6—C4—N4—Cd2	2.7 (3)	C13—C4—N6—N5	155.5 (2)
C6—C5—N4—C4	−0.4 (4)	C5—C6—N7—C13	1.0 (5)
C6—C5—N4—Cd2	175.7 (2)	C5—C6—N7—Cd1^v^	−176.2 (2)
N1^iv^—Cd2—N4—C4	37.2 (3)	C4—C13—N7—C6	−0.5 (4)
N3—Cd2—N4—C4	114.9 (2)	C4—C13—N7—Cd1^v^	176.70 (19)
N5—Cd2—N4—C4	9.15 (18)	N1^iv^—Cd2—S1—C9	113.39 (13)
S1—Cd2—N4—C4	−151.14 (18)	N3—Cd2—S1—C9	20.49 (14)
S3—Cd2—N4—C4	−69.38 (19)	N5—Cd2—S1—C9	−109.59 (17)
N1^iv^—Cd2—N4—C5	−138.9 (3)	N4—Cd2—S1—C9	−63.61 (13)
N3—Cd2—N4—C5	−61.2 (3)	S3—Cd2—S1—C9	−155.29 (12)
N5—Cd2—N4—C5	−166.9 (3)	N2—Cd1—S2—C8	−7.34 (12)
S1—Cd2—N4—C5	32.8 (2)	N2^i^—Cd1—S2—C8	172.66 (12)
S3—Cd2—N4—C5	114.6 (2)	N7^ii^—Cd1—S2—C8	78.70 (11)
C2—C1—N5—N6	0.7 (3)	N7^iii^—Cd1—S2—C8	−101.30 (11)
C2—C1—N5—Cd2	144.6 (2)	N1^iv^—Cd2—S3—C7	−19.29 (12)
N1^iv^—Cd2—N5—C1	28.0 (3)	N5—Cd2—S3—C7	73.89 (11)
N3—Cd2—N5—C1	120.4 (3)	N4—Cd2—S3—C7	141.13 (11)
N4—Cd2—N5—C1	−161.5 (3)	S1—Cd2—S3—C7	−124.53 (11)

Symmetry codes: (i) −*x*+1, −*y*, −*z*; (ii) *x*+1, *y*−1, *z*; (iii) −*x*, −*y*+1, −*z*; (iv) −*x*+1, −*y*+1, −*z*+1; (v) *x*−1, *y*+1, *z*.
